# Surgical Management of Recurrent Retroperitoneal Sarcomas: Experience From a High‐Volume Sarcoma Centre: Recurrent Retroperitoneal Sarcomas: Outcomes of Multiple Recurrences

**DOI:** 10.1002/jso.70264

**Published:** 2026-05-06

**Authors:** Abdeali Saif Arif Kaderi, Tanvi M. Shah, Shraddha Patkar, Y. Aditya Sai Ram, Vinayaka S. Bairannavar, M. Myvizhi Kannan, Bharat Rekhi, Prabhat Bhargav, Kunal B. Gala, Afroj Bagwan, Mukta Ramadwar, Jifmi Jose, Nehal Khanna, Siddhartha Laskar, Mahesh Goel

**Affiliations:** ^1^ Department of Surgical Oncology Tata Memorial Hospital, Homi Bhabha National Institute Mumbai Maharashtra India; ^2^ Division of Hepaobiliary, Gastrointestinal and Retroperitoneal Sarcoma surgery, Department of Surgical Oncology Tata Memorial Hospital, Homi Bhabha National Institute Mumbai Maharashtra India; ^3^ Department of Pathology Tata Memorial Hospital, Homi Bhabha National Institute Mumbai Maharashtra India; ^4^ Department of Medical Oncology Tata Memorial Hospital, Homi Bhabha National Institute Mumbai Maharashtra India; ^5^ Department of Interventional Radiology ACTREC, Homi Bhabha National Institute Mumbai Maharashtra India; ^6^ Department of Radiotherapy Tata Memorial Hospital, Homi Bhabha National Institute Mumbai Maharashtra India

**Keywords:** compartmental resections, multiple recurrences, recurrent retroperitoneal sarcomas, retroperitoneal sarcomas

## Abstract

**Background:**

Retroperitoneal sarcoma (RPS) is a rare and complex malignancy, requiring specialized multidisciplinary care. While a significant progress has been made in managing a primary RPS, there is a limited literature on the outcomes of recurrent RPS (RecRPS). This study evaluates the oncological outcomes of RecRPS at a leading sarcoma referral center in India.

**Methods:**

A retrospective analysis was performed for patients with RecRPS who underwent surgery between January 2011 and December 2024. Clinical outcomes were analyzed using Kaplan‐Meier method and compared using log‐rank test. Extended Cox regression models were used to account for intra‐individual correlation, in cases of multiple recurrence. Prentice, William and Peterson model (PWP) CP model (total time) and PWP Gap time (PWP‐GT) models were employed to estimate predictors of multiple recurrences.

**Results:**

Out of 285 patients with primary RPS, 160 (56.1%) underwent surgery for a recurrent disease. The median overall survival (OS) was 137.1 months for the entire cohort and 41.92 months in RecRPS. The median OS of patients with 1st recurrence was 38.97 months. For subsequent recurrences, the median OS were 74.94 months (2nd recurrence), 57.4 months (3rd recurrence) and 54.2 months (4th recurrence), respectively. The various clinicopathological parameter associated with multiple recurrences were R+resection or resection with unknown margins, dedifferentiated liposarcoma and leiomyosarcoma, as histopatholologic subtypes; histologic organ invasion (HOI) of the small bowel and vessels, requirement of adjuvant and neoadjuvant therapy, disease progression on neoadjuvant chemotherapy and grade IIIb and IV complications.

**Conclusion:**

While the best chance of cure is at the primary presentation, some patients may experience prolonged disease control even with multiple recurrence, if treated optimally.

## 1 Introduction

The recognition of retroperitoneal sarcoma (RPS) surgery as a sub‐speciality has led to the establishment of multiple international as well national level forums, in the form of international tumor boards and institutional multidisciplinary tumor board meetings (MDTs), to guide and standardize the treatment of these relatively complex tumors [1]. While newer milestones are being reached in improving the outcomes, following pioneering studies such as STRASS [2] and the ongoing STRASS‐2 [3], providing randomized evidence for a primary RPS, there remains a notable gap in the literature on recurrent RPS and their treatment outcomes. A few retrospective studies have demonstrated the benefit of surgery in RecRPS, especially for the initial two recurrences, however it is difficult to draw conclusive evidence owing to their small sample sizes and heterogeneity [4]. It is therefore crucial to audit the outcomes of RecRPS in order to understand the efficacy of our interventions. This study was aimed to analyze the outcomes of RecRPS treated at a high‐volume sarcoma referal center from India.

## 2 Materials and Methods

### 2.1 Study Design, Setting and Patients

A retrospective analysis of a prospectively maintained database of patients, who underwent surgery for RPS in the adult RPS services of Tata Memorial Hospital, Mumbai, from January 2011 to December 2024, was performed. Out of this entire cohort, patients who underwent surgical resection for recurrent disease were identified and their outcomes were analyzed. Preoperative radiological findings; intraoperative findings and histological reports including details of histologic organ invasion (HOI) were noted. Postoperative complications, graded by the Clavien‐Dindo (CD) classification system and duration of hospital stay were also recorded [5].

### 2.2 Treatment

**Figure 1:**
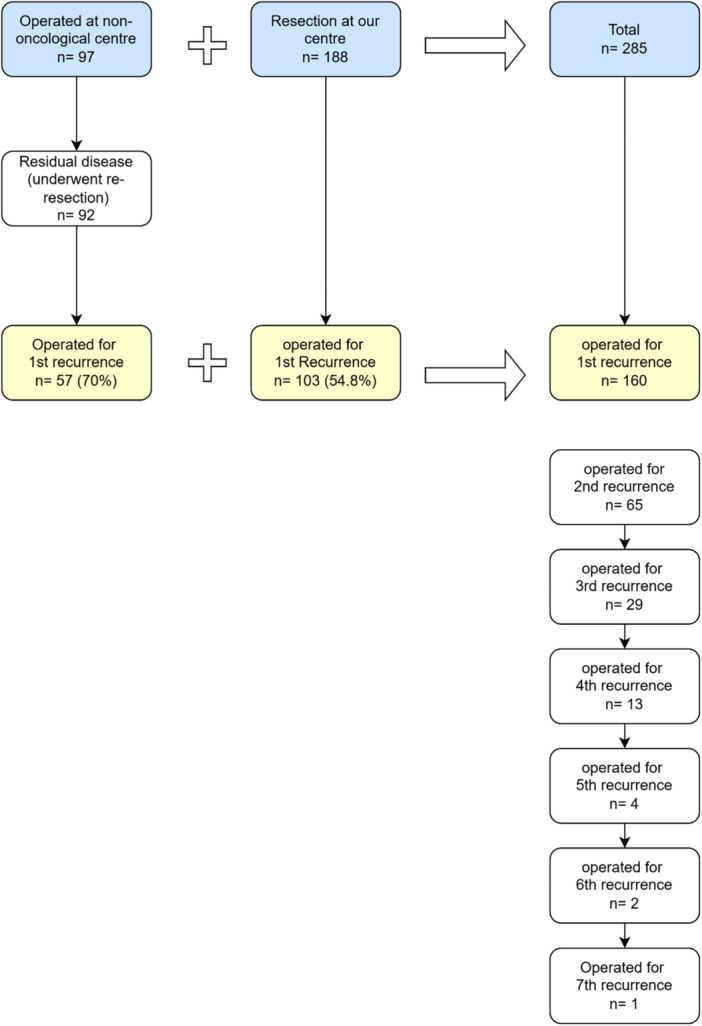
Total number and distribution of patients with primary and recurrent retroperitoneal sarcomas in the cohort.

All RPS patients were discussed in the multidisciplinary RP tumor board, including sarcoma surgeons, radiologist, radiation oncologist, medical oncologist and a pathologist. Surgical plans were made based on the radiological findings and the histopathological evaluation. Most patients, except for those with characteristic imaging findings, with a primary RPS underwent a preoperative biopsy. For patients who presented with a biopsy that was performed elsewhere, or resections done elsewhere, the biopsy and/or the corresponding histopathology slides and blocks were reviewed. Patients referred after surgery at non‐oncological center underwent an initial assessment with contrast enhanced computed tomography (CECT) and the status was characterized as having residual disease versus no residual disease. Patients with residual disease underwent immediate re‐resection of residual disease (i.e not surgery for recurrence) and then entered surveillance. A subsequent recurrence for these patients was classified as first recurrence [Figure 1]. Grading was done by the French Federation of Cancer Centers Sarcoma Group (FNCLCC) system [6]. Neoadjuvant chemotherapy (NACT) was planned based on MDT discussion in patients with high grade sarcomas where upfront surgery was expected to be morbid and with the aim to evaluate the disease biology before proceeding to extensive multi‐visceral resections. A diethylenetriamine pentaacetate (DTPA) renogram was performed for all patients planned for compartmental resection. Adjuvant therapy were based on the final histopathology and customized as per previous lines of chemotherapy received. Adjuvant radiation therapy (RT) was used selectively in cases of R+ resections. Follow‐up was initially performed 3‐monthly for 2 years, 6‐monthly for the next 3 years, and annually thereafter. Ultrasonography of the abdomen, an erect X‐ray chest, alternating with CECT and renal function tests were performed at each follow‐up.

### 2.3 Definitions

Surgical procedures were classified as follows:

1. Compartment resections, defined as en bloc resection of organs within 1–2 cm of the tumor surface, including diaphragm in order to obtain a rim of the normal tissue all around.
2. en bloc resections with involved organs
3. Tumor excisions alone; and
4. 4) Vascular resections (defined as resection of major vessels, i.e. inferior vena cava [IVC] and/or abdominal aorta with or without reconstruction) [7, 8].

A dedicated MDT for RPS was established at our Institution in the year 2018. Accordingly, this cohort was divided into pre‐MDT era (January 2011 to December 2017) and post‐MDT era (January 2018 to December 2024).

### 2.4 Outcome Measures

The median overall survival (OS) was calculated from the date of index surgery (including the index surgery done at our center, or the index surgery done elsewhere and subsequently operated at our center for residual disease) until the last follow‐up or death. Factors affecting survival in recurrent RPS and factors associated with multiple recurrences were evaluated using multivariable models.

Statistical Methods:

Descriptive statistics were employed to summarize the baseline characteristics of patients. Continuous variables were reported as the mean ± standard deviation (SD), while categorical variables were expressed as frequencies and percentages. primary and recurrent RPS groups were compared in terms of histopathologic subtypes, completeness of resection, HOI, margin status, complications, organ resections, and the use of adjuvant and neoadjuvant treatments, using the chi‐square test or Fisher's exact test as appropriate. OS, along with the survival probability rates at specific time points, were estimated using the Kaplan‐Meier method. An extended Cox model was employed to account for intra‐individual correlation in cases of multiple recurrences after retroperitoneal sarcoma surgery. To estimate predictors of subsequent recurrences, the Prentice William and Peterson model (PWP), CP model (total time) and PWP Gap time (PWP‐GT) models were employed [9]. These extended models were used instead of Cox proportional Hazard model, since here the subsequent events (recurrences) are a function of a previous event (previous recurrence). The univariate and multivariate analyses were done to estimate the hazard ratios (HR) using the aforementioned extended Cox models, along with 95% confidence intervals (CI). Predictors for the multivariate model were selected based on univariate outputs, with a *p*‐value of ≤ 20%. For the multivariate analysis, all statistical tests were two‐sided, and a *p*‐value of less than 0.05 was deemed statistically significant. The statistical analyses were performed using STATA 17 and R version 4.3.3.

### 2.5 Ethics

The data collection was in accordance with the standards of 1964 Helsinki Declaration and its latest amendment (2013). The study protocol was approved by institutional ethics committee (Project Number 901186)

## 3 Results

A total of 285 patients with primary RPS were screened from the institutional database. These included 188 patients, who underwent primary surgery at our center, while 97 were referred after previous inadequate surgery done at a non‐oncological center and subsequently underwent a definitive adequate surgery (for a residual disease) at our center. Subsequently, a total of 160 (56.14% of the total 285) patients, who underwent surgery for 1st recurrence, were identified and included in final analysis. Further follow‐up data revealed, 65 of these patients were operated for second recurrence, 29 patients for third recurrence, 13 patients for fourth recurrence, 4 patients for 5th recurrence, 2 patients for sixth recurrence and 1 patient for 7th recurrence. Figure 1.

### 3.1 Demographic Variable

The median age of the patients was 52 years (range 17–77 years). The demographic details of the cohort are presented in Table 1. The recurrent disease group included 92 (57.5%) male and 68 (42.5%) female patients. (Table 1).

**Table 1:** Patients baseline characteristics.

		Total(*n* = 285)	No recurrence (*n* = 125)	Recurrence (*n* = 160)	*p*‐value
Gender	Male	170 (59.6)	78 (62.4)	92 (57.5)	0.403
	Female	115 (40.4)	47 (37.6)	68 (42.5)	
Site	RP	234 (82.1)	97 (77.6)	137 (85.6)	0.198
	Pelvis	17 (6.0)	10 (8.0)	7 (4.4)	
	Vascular	34 (11.9)	18 (14.4)	16 (10.0)	
Resection status	R0	188 (66.0)	105 (84.0)	83 (51.9)	< 0.001
	R+	45 (15.8)	17 (13.6)	28 (17.5)	
	Unknown	52 (18.2)	3 (2.4)	49 (30.6)	
Histology	WDLPS	67 (23.5)	33 (26.4)	34 (21.2)	0.091
	DDLPS	92 (32.3)	33 (26.4)	59 (36.9)	
	LMS	76 (26.7)	31 (24.8)	45 (28.1)	
	Others	50 (17.5)	28 (22.4)	22 (13.8)	
HOI: Small bowel	No	262 (91.9)	120 (96.0)	142 (88.8)	0.026
	Yes	23 (8.1)	5 (4.0)	18 (11.2)	
HOI: Colon	No	232 (81.4)	104 (83.2)	128 (80.0)	0.491
	Yes	53 (18.6)	21 (16.8)	32 (20.0)	
HOI: Kidney	No	247 (86.7)	106 (84.8)	141 (88.1)	0.413
	Yes	38 (13.3)	19 (15.2)	19 (11.9)	
Margin status	Negative	241 (84.6)	112 (89.6)	129 (80.6)	0.012
	Positive	38 (13.3)	9 (7.2)	29 (18.1)	
	NA (in‐op)	6 (2.1)	4 (3.2)	2 (1.2)	
Adjuvant Treatment	None	136 (47.7)	72 (57.6)	64 (40.0)	0.001
	Adjuvant systemic therapy	79 (27.7)	35 (28.0)	44 (27.5)	
	Adjuvant radiation	70 (24.6)	18 (14.4)	52 (32.5)	
Neoadjuvant therapy	No	234 (82.1)	97 (77.6)	137 (85.6)	0.079
	Yes	51 (17.9)	28 (22.4)	23 (14.4)	
Response to NACT	Partial	11 (3.9)	6 (4.8)	5 (3.1)	0.118
	Stable	35 (12.3)	21 (16.8)	14 (8.8)	
	Progression	5 (1.8)	1 (0.8)	4 (2.5)	
	NA	234 (82.1)	97 (77.6)	137 (85.6)	
MDT	Pre	81 (28.4)	19 (15.2)	62 (38.8)	< 0.001
	Post	204 (71.6)	106 (84.8)	98 (61.3)	
HOI: Pancreas	No	272 (95.4)	120 (96.0)	152 (95.0)	0.688
	Yes	13 (4.6)	5 (4.0)	8 (5.0)	
HOI: Duodenum	No	277 (97.2)	121 (96.8)	156 (97.5)	0.733
	Yes	8 (2.8)	4 (3.2)	4 (2.5)	
HOI: Vascular	No	268 (94.0)	112 (89.6)	156 (97.5)	0.005
	Yes	17 (6.0)	13 (10.4)	4 (2.5)	
Complications	0‐II	248 (87.0)	113 (90.4)	135 (84.4)	0.425
	IIIa	10 (3.5)	4 (3.2)	6 (3.8)	
	IIIb	17 (6.0)	4 (3.2)	13 (8.1)	
	IV	6 (2.1)	3 (2.4)	3 (1.9)	
	V	4 (1.4)	1 (0.8)	3 (1.9)	

### 3.2 Surgery

The primary surgeries included 128 (44.9%) compartment/multi‐visceral resections, 72 (25%) en‐bloc resections, 42 (14.6%) tumor excisions, 27 (9.4%) vascular resections and 10 (3.5%) debulking (palliative) surgeries. Among the 160 recurrent surgical resections, the number and percentage of compartment/multi‐visceral resections, en‐bloc resections, tumor excisions, vascular resections and debulking (palliative) surgeries were 61 (38.1%), 42 (26.3%), 28 (17.5%), 18 (11.3%) and 9 (5.6%), respectively. Four patients with primary and 2 with recurrent disease were found to be inoperable at exploration.

### 3.3 Histopathologic Subtypes

The most common histopathologic subtype was well‐differentiated liposarcoma (WD‐LPS) (34, 21.3%) followed by de‐differentiated liposarcoma (DD‐LPS) (59, 36.9%), and LMS (45, 28.1%), in the recurrent settings. There was a similar pattern of histopathologic subtypes in the primary RPS. The less common subtypes (22, 13.8%) included solitary fibrous tumors, pleomorphic sarcomas, malignant peripheral nerve sheath tumors, angiosarcomas, synovial sarcomas, myofibroblastic sarcomas and follicular dendritic cell sarcomas among the RecRPSs. [Table 1].

Histologic organ invasion (HOI)

Histologic organ invasion (HOI), defined as infiltration of the organ parenchyma or a vessel wall by tumor, was seen in 135 (47.36%) patients in the primary setting (Table 1). The small bowel was more commonly involved in the recurrent group, compared to the primary (*p* = 0.026) [Table 1]. The number of HOI in patients with nil to 7 recurrences are summarized in the supplementary Table 1.

Neoadjuvant chemotherapy (NACT) and Adjuvant chemotherapy:

NACT was given to 28 (22.4%) patients with primary disease and 23 (14.4%) patients with RecRPS. Adjuvant systemic therapy was given to 79 patients in primary disease and 44 patients in the RecRPS settings. Adjuvant RT was given to 70 patients in primary and 52 patients in RecRPS settings. A significantly higher proportion of patients received adjuvant therapy in RecRPS (*p* value 0.001).

### 3.4 Outcomes

Intraoperative and Post‐Operative Outcomes

The median blood loss was 1500 ml and median hospital stay was 8 days (Range: 2–65 days for primary and 1–49 days for RecRPS) for both primary and RecRPS. The post‐operative outcomes scaled using the Clavian‐Dindo scale (CD) were also not significantly different between primary and recurrent RPS. [Table 1] R0 resection rates were significantly higher in the primary versus the recurrent group (*p* < 0.001). Six patients (3.8%) had CD grade IIIa complications, 13 (8.1%) had grade IIIb complications. Three patients (1.9%) had CD grade IV and grade V complications and there were no significant differences in grade 3‐4 complications between the groups.

### 3.5 Long Term Outcomes

#### 3.5.1 Overall Survival (OS)

The median duration of follow‐up of the whole cohort was 48.19 months (95% CI 39.70–56.68 months) calculated by using the reverse KM curve, while that of the RecRPS was 58.02 months (95% CI 49.35–66.69 months).

The median OS of 285 patients was 58.3 months (95%CI 42.8–73.4) and the 3‐year OS was 62.9 (95%CI 55.8–69.1). The median OS was 137.1 months in primary versus 41.92 months in RecRPS with a *P* value of 0.001.

**Figure 2:**
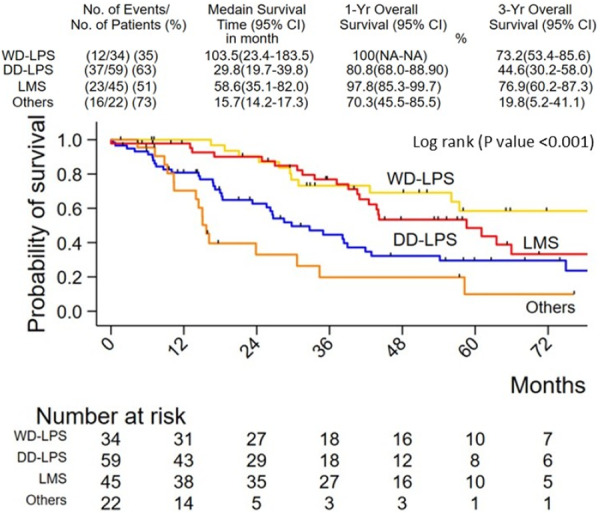
Kapla Meir curves of overall survival (OS), stratified by histologies in the patients with recurrent retroperitoneal sarcomas (RecRPS).

The median OS in cases of primary WDLPS was not reached compared to median OS of Recurrent WDLPS of 74.2 months (46.8–101.5months); *p* = 0.063. The median OS of primary DDLPS was 30.3 (19.7–40.9) months while that of recurrent DDLPS was 38.1 months (46.22.1–54.2); *p* = 0.983. The median OS of primary LMS was 63.5 months (40.1–86.9) and that of recurrent LMS was 58.6 months (31.5–85.7); *p* = 0.821 [Figure 2].

The median OS of patients with Rec1 was 38.97 months, those with Rec2 were 74.94 months, for Rec3 patients, it was 57.4 months and for Rec4 patients, it was 54.2 months.

**Figure 3:**
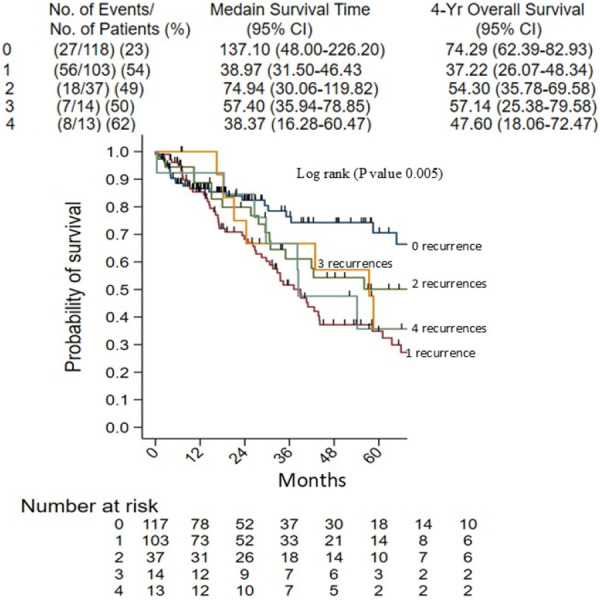
Kaplan‐Meir curves for overall survival of patients with recurrent retroperitoneal sarcomas stratified by number of recurrences.

The survival differences in patients with multiple recurrences are illustrated in Figure 3. With reference to no recurrence group, the HR for survival for patients with multiple recurrences were 2.36 (CI 1.50–3.72; *p* < 0.001) for patients with one recurrence; 1.54 (CI 0.84–2.82; *p* = 0.159) for those with second recurrence, 1.69 (CI 0.76–3.72; *p* = 0.192) for third recurrence and 1.50 (CI 0.65–3.44; *p* = 0.337) for 4th recurrence.

#### 3.5.2 Prognostic Factors for Survival in RecRPS

**Figure 4:**
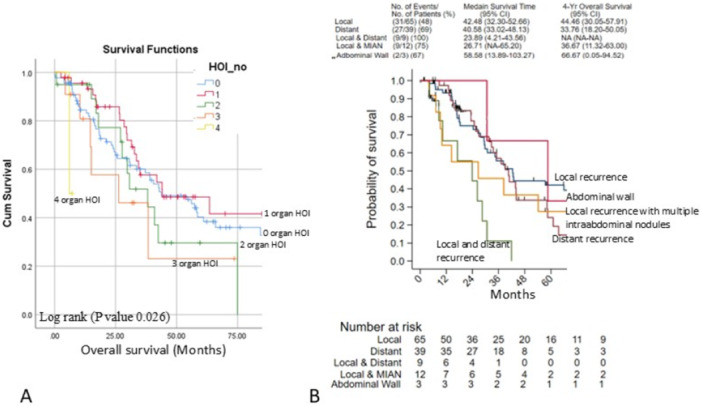
(A) Kaplan Meir curves (KM) for overall survival (OS) of patients with recurrent retroperitoneal sarcomas (RecRPS) stratified by number of organs with histologic invasion (HOI). HOI 0: no histologic invasion. HOI 1, 2, 3, 4: Histologic organ invasion of 1, 2,3 and 4 organs, respectively. (B) KM curves for OS of patients with RecRPS stratified by pattern of recurrence.

Among RecRPS, the increase in the number of organs with HOI was associated with a significantly worse OS (*p* = 0.02) The median OS in patients with no or a single HOI was 43.76 months and 44.15 months, respectively, while it dropped to 26.2 months for 3 HOI and was much lower, i.e. 6.1 months in patients with 4 HOIs (Figure 4).

Patterns of multiple recurrences:

One hundred and two (63.75%) patients had local recurrences, 35 (21.9%) had distant recurrences, while 9 (5.6%) had local, as well as distant recurrences. Recurrence over the abdominal wall was seen in 3 (1.9%) patients. Intra‐abdominal sarcomatosis was seen in 11 (6.9%) patients. The median OS in patients with local recurrence was 42.6 months, and for distant recurrence was 33 months, for patients with local as well as distant recurrences, the median OS was 23.9 months and for those with sarcomatosis was 26.7 months. The patients with abdominal wall recurrence had an OS of 58 months (Figure 4b).

#### 3.5.3 Factors Predicting Risk of Multiple Recurrences

On univariate analysis, an incomplete resection (R+), a margin positive resection, small bowel HOI, vascular involvement as HOI and the need for adjuvant and neoadjuvant therapies were found to be associated with risk of multiple recurrences. Multiple recurrences were significantly lower in post MDT era and if the treatment was done in oncological institute.

Multivariate analysis of multiple recurrences was performed using the PWP‐CP model (total time) and PWP Gap time (PWP‐GT) models [9]. According to the PWP‐CP model, R+ resection, DDLPS and LMS histopathologic subtypes, involvement of small bowel, requirement of adjuvant and neoadjuvant therapy, disease progression on NACT and grade IIIb and IV complications resulted in higher recurrences, while unknown margin status, requirement of adjuvant radiation and vascular involvement as HOI resulted in higher recurrence rates, as per PWP‐GT model (Table 2).

**Table 2:** Univariate and multivariate analysis using the PWP‐total time model and PWP‐gap time model.

		PWP‐total time model				PWP‐gap time model			
		Unadjusted HR (95% CI)	*p*‐value	Adjusted HR (95% CI)	*p*‐value	Unadjusted HR (95% CI)	*p*‐value	Adjusted HR (95% CI)	*p*‐value
Gender	Male	1 (Ref)		—	—	1 (Ref)		—	—
	Female	1.00 (0.79–1.28)	0.980	—	—	1.02 (0.79–1.32)	0.877	—	—
Site	RP	1 (Ref)		—	—	1 (Ref)		—	—
	Pelvis	0.62 (0.38–1.01)	0.055	—	—	0.63 (0.39–1.01)	0.055	—	—
	Vascular	0.94 (0.65–1.36)	0.735	—	—	0.97 (0.67–1.41)	0.868	—	—
Resection status	R0	1 (Ref)		1 (Ref)		1 (Ref)		1 (Ref)	
	R+	1.51 (1.03–2.22)	**0.034**	1.30 (0.85–1.98)	0.231	1.42 (0.96–2.09)	0.075	1.28 (0.83–1.97)	0.272
	Unknown	1.25 (0.95–1.64)	0.119	1.21 (0.88–1.67)	0.245	1.22 (0.91–1.65)	**0.187**	1.28 (0.94–1.76)	0.116
Adjuvant received at index surgery	No	1 (Ref)		1 (Ref)		1 (Ref)		1 (Ref)	
	Yes	1.20 (0.94–1.55)	0.151	0.90 (0.64–1.28)	0.558	1.22 (0.94–1.59)	0.136	0.9 (0.64–1.26)	0.533
	Unknown	1.76 (0.91–3.41)	0.092	1.16 (0.54–2.47)	0.699	1.60 (0.90–2.82)	0.107	1.02 (0.53–1.98)	0.950
Histology	WDLPS	1 (Ref)		1 (Ref)		1 (Ref)		1 (Ref)	
	DDLPS	1.86 (1.33–2.62)	**< 0.001**	1.85 (1.25–2.76)	**0.002**	1.71 (1.24–2.36)	0.001	1.69 (1.18–2.41)	**0.004**
	LMS	1.52 (1.08–2.15)	**0.016**	1.61 (1.10–2.36)	**0.013**	1.45 (1.05–2.02)	0.026	1.49 (1.04–2.14)	**0.031**
	Others	1.19 (0.76–1.87)	0.456	1.12 (0.70–1.79)	0.635	1.09 (0.70–1.70)	0.704	1.02 (0.64–1.62)	0.943
HOI: Small bowel	No	1 (Ref)		1 (Ref)		1 (Ref)		1 (Ref)	
	Yes	1.53 (1.09–2.15)	**0.014**	1.41 (0.96–2.07)	0.081	1.39 (0.95–2.03)	0.088	1.23 (0.80–1.87)	0.346
HOI: Colon	No	1 (Ref)		—	—	1 (Ref)		—	—
	Yes	1.10 (0.85–1.42)	0.468	—	—	1.21 (0.92–1.59)	0.180	—	—
HOI: Kidney	No	1 (Ref)		—	—	1 (Ref)		—	—
	Yes	0.82 (0.53–1.26)	0.359	—	—	0.80 (0.55–1.17)	0.253	—	—
Margin status	Negative	1 (Ref)		—	—	1 (Ref)		—	—
	Positive	1.25 (0.91–1.71)	0.172	—	—	1.13 (0.81–1.58)	0.482	—	—
	NA (in‐op)	1.30 (0.37–4.55)	0.685	—	—	1.28 (0.36–4.47)	0.705	—	**—**
Adjuvant Treatment	None	1 (Ref)		1 (Ref)		1 (Ref)		1 (Ref)	
	Adjuvant systemic therapy	1.56 (1.15–2.11)	**0.004**	1.49 (1.02–2.19)	**0.041**	1.65 (1.21–2.24)	0.001	1.49 (1.03–2.17)	**0.037**
	Adjuvant radiation	1.23 (0.91–1.67)	0.178	1.39 (0.95–2.05)	0.093	1.28 (0.95–1.73)	**0.108**	1.36 (0.95–1.95)	0.088
Neoadjuvant therapy	No	1 (Ref)		1 (Ref)		1 (Ref)		1 (Ref)	
	Yes	1.60 (1.05–2.44)	**0.028**	1.53 (1.00–2.33)	0.050	1.62 (1.07–2.44)	**0.021**	1.45 (0.96–2.20)	0.077
Response to NACT	Partial	1 (Ref)		—	—	1 (Ref)		—	—
	Stable	1.54 (0.66–3.59)	0.321	—	—	1.48 (0.62–3.52)	0.376	—	—
	Progression	2.61 (1.06–6.41)	**0.036**	**—**	**—**	2.40 (1.06–5.40)	0.035	**—**	—
	NA	0.99 (0.49–2.01)	0.978	—	—	0.95 (0.46–1.97)	0.898	—	—
MDT	Pre	1 (Ref)		—	—	1 (Ref)		—	—
	Post	0.91 (0.71–1.16)	0.432	—	—	0.98 (0.75–1.28)	**0.899**	—	**—**
HOI: Pancreas	No	1 (Ref)		—	—	1 (Ref)		—	—
	Yes	0.90 (0.51–1.58)	0.710	—	—	0.91 (0.54–1.56)	0.741	—	—
HOI: Duodenum	No	1 (Ref)		—	—	1 (Ref)		—	—
	Yes	1.27 (0.85–1.89)	0.243	—	—	1.23 (0.84–1.79)	0.289	—	—
HOI: Vascular	No	1 (Ref)		—	—	1 (Ref)		—	—
	Yes	0.64 (0.24–1.73)	0.380	—	—	0.64 (0.24–1.72)	**0.377**	—	**—**
Complications	0–II	1 (Ref)		1 (Ref)		1 (Ref)		1 (Ref)	
	IIIa	1.23 (0.58–2.64)	0.589	1.85 (0.84–4.09)	0.128	1.11 (0.46–2.64)	0.819	1.67 (0.72–3.83)	0.231
	IIIb	1.33 (0.87–2.01)	0.186	1.66 (1.04–2.66)	**0.034**	1.07 (0.67–1.71)	0.762	1.28 (0.77–2.11)	0.341
	IV	1.68 (1.14–2.47)	**0.008**	2.19 (1.25–3.84)	**0.006**	1.69 (1.15–2.49)	0.008	1.98 (1.26–3.11)	**0.003**
	V	0.94 (0.57–1.57)	0.827	0.76 (0.39–1.48)	0.423	0.54 (0.32–0.92)	0.022	0.47 (0.25–0.86)	**0.014**

*Note:* Bold values indicate statistical significant at *p* < 0.05.

### 3.6 Impact of Center of Treatment

Ninety‐two out of 97 (93.9%) patients who underwent surgery at a non‐oncology center had residual disease. After re‐surgery (for residual disease), 57 out of these 92 patients (70%) developed recurrence. In contrast, 103 out of 188 (54.78%) patients operated at the oncological (our) center developed recurrence, A significant disease free survival (DFS) difference was observed between surgery done at oncological center, as compared to inadequate surgery performed elsewhere (median DFS: 33.54 months versus 14.59 months, *p* < 0.001). Additionaly, within the center, tumor recurrences were significantly lower in the post MDT era. (*p* < 0.001), (Table 1).

## 4 Discussion

The principles of surgical management of primary retroperitoneal sarcomas have evolved over the time from simple excisions, to compartmental resections [10] and histopathology‐based tailored approaches [11]. Standardization of procedures and centralization of care have led to a significant improvement in the survival outcomes [12, 13]. However, recurrences, often multiple recurrences, still remain a challenge. While the role of surgical resection in RecRPS has also been studied in previously [14, 15], standard principles are lacking with respect to the patient selection for aggressive re‐resections for multiple recurrences.

In this retrospective cohort, 160 patients underwent surgery for at least a single recurrence, while some of those underwent surgical resection even up to 4th recurrences. The cumulative median OS in the recurrent group was an acceptable 41 months. This clearly justifies the role of surgery for RecRPS.

The OS was excellent in both primary (median OS not reached) and recurrent WD‐LPS. (74 months) suggestive of the biologically indolent nature of these tumors. The spectrum of management of these tumors, therefore, ranges from active surveillance to aggressive re‐resections. Singer et al. suggested a variability in the prognosis based on the growth rate more than 0.98 cm [12]. Another study that included both, RPS and limb saromas revealed a higher grade and growth rate of exceeding 0.68 cm to be associated with a higher disease‐specific death [14]. Nomograms have been devised and externally validated to predict the outcomes of surgery for first recurrence, aiding in planning of active surveillance [16, 17]. Nevertheless, the excellent survival outcomes seen in the patients with recurrent WD‐LPS in our series consolidate the role of aggressive re‐resections in well selected patients harboring these tumors.

In patients with DD‐LPS and LMS, the median OS was comparable between the primary and the recurrent disease group, and was as high as 58 months, for recurrent LMS. Contemporary studies show a higher decline in 3 and 5 year OS of recurrent LMS, compared to primary LMS, and their specific recurrence rate exceed the values of that of other histologies [18]. This difference in pattern seen in LMS is due to its higher propensity for distant metastasis [18]. Conventionally, high‐grade sarcomas are considered to be biologically aggressive, however our findings signify the role of surgery for recurrent disease (along with optimal systemic therapy) even in these histopathologic subtypes [1]. This is likely due to better patient selection and optimal utilization of perioperative systemic therapy.

In well‐selected patients in our cohort, an acceptable OS exceeding 50 months was seen in even up to 4 recurrences. While the numbers of these patients are small, nevertheless the promising OS gives an opportunity to explore those subgroups of patients who can benefit from surgery even after multiple recurrences. Notably, the hazard ratio for survival steeply dropped down at the 1st recurrence. This re‐consolidates the basic principle of sarcoma surgery that the 1st surgery, done adequately, is the best chance of cure. Strikingly, the hazard ratios for survival, showed no significant difference between the patients who had a single recurrence, compared to those who have subsequent recurrences. This is in contrast to previous studies. Wang et. al. suggested equivalent outcomes of primary resection and RecRPS1 surgery and worsened outcomes with RecRPS2 and beyond [19]. A probable reason suggested by the authors was that the patients with 2 or more recurrence had higher grade tumors and macroscopically incomplete resection. A contemporary study reports a surgery free survival of 25.9 months, which tended to decrease with subsequent resections [20]. In context of our retrospective study, the finding of no significant difference is multi‐factorial. Firstly, it potentially suggests better disease biology (patients who continue to present with recurrences that are resectable, probably have less aggressive disease biology). Secondly, it is mostly likely due to stringent patient selection in the setting of an MDT. Overall, within the limitation of a retrospective cohort, this finding consolidates the role of surgery even for multiple recurrences, in well selected patients.

As we emphasize that patient selection remains the key for optimal outcomes, we sought to develop multivariate models to predict factors associated with development of multiple recurrences. Unlike the Cox regression models which assume proportional hazard risks, we used the PWP and PWP‐GT models, to negate the confounding effect of the previous recurrences. These models would in turn guide us in better patient selection.

For example, in our cohort, four or more organs with HOI correlated with a sudden decrease of OS from 26.3 months to 6.1 months in three HOI patients. More number of organs removed per resection correlated with a worse prognosis as per a study by TARPSWG group [16]. Therefore, while preoperative planning, if four or more organs are contemplated to be infiltrated/encased [21], there is a possibility of a futile resection. Based on our findings, we predict the following patients to have higher recurrence risks and therefore may be poor candidates for surgery in recurrent setting:

1. Complete resection seems unfeasible
2. Small bowel involvement
3. Vascular involvement
4. Multiple (4 or more) organs are involved
5. Progression of neoadjuvant therapy
6. Contemplated morbid resections

The pattern of recurrence also determines the outcomes and can therefore guide patient selection. Patients with recurrences only in their abdominal wall have an excellent survival (median of more than 50 months), while the patients with abdominal sarcomatosis have a much lower survival. Likewise, Jolissaint et. al. reported that intraabdominal multifocality of the tumor portends a poor prognosis and defined a cut‐off of seven foci, to call off curative surgery [22].

In our cohort, 92 out of 97 (93%) patients who underwent completion surgery at our center (after being referred with an incomplete resection elsewhere) had residual disease, even after a reported adequate margin‐negative initial surgery. Contrastingly, 103 out of 188 (54%) patients, who underwent a primary surgery at our center, developed tumor recurrences. This re‐emphasizes the significance of centralization of care to specialized sarcoma centers for better outcomes [10, 11]. Also, within the center, the recurrences significantly decreased after the establishment of a dedicated sarcoma tumor board. This may be due to better patient selection, better adoption of adjuvant and neoadjuvant treatments and standardization of the surgical procedures.

This is a single‐center study from a high‐volume cancer referral sarcoma center in the country, with standardized, evidence‐based management protocols and surgeries performed by specialized sarcoma surgeons. This study establishes that judicious surgical resection leads to meaningful survival outcomes even in the patients with multiple recurrences. It establishes a broad framework for patient selection for aggressive surgical management, using robust statistical methods.

Nevertheless, there are several limitations. Given this constitutes a retrospective cohort, the outcomes are heavily driven by an inherent selection bias and might not be a true representation of the general cohort of patients with recurrent RPS, a vast majority of whom are not candidates for re‐resections. The patients who had distant recurrences and those with local recurrences who were either kept on surveillance or deemed with unresectable tumors were not included. Consequently, the real denominator was challenging to capture. Also, for a rare and heterogenous disease entity such as RPSs, the number of patients, especially in the multiple recurrence group and rare histopathological subtypes from any single center, will always be lesser to establish high‐level evidence. Furthermore, quality of life outcomes have not been recorded and studied in this study. Nevertheless, the study establishes the feasibility of such research and calls for international collaborations for multi‐center studies, which could better establish the management principles of recurrent RPSs.

## 5 Conclusion

This study constitutes one of the largest cohort of its kind from Indian subcontinent. While the best chance of cure is at the time of primary presentation, some patients may experience prolonged disease control even with multiple recurrence, if treated optimally. Therefore, re‐operative surgeries for multiple recurrences should be considered worthwhile in well‐selected patients.

## Author Contributions

S.P., M.G. and A.S.A.K. conceptualized the study. A.S.A.K., T.M.S. and S.P. wrote the initial manuscript. V.S.B., Y.A.S.R., A.S.A.K. and A.B. did the data collection. M.K.M. and A.S.A.K. did the data analysis. All authors contributed to the rigorous scrutiny of the manuscript accuracy. All authors have approved the final version of the manuscript.

## Funding

The authors have nothing to report.

## Ethics Statement

The study protocol was approved by the institutional ethics committee (Project Number 901186).

## Conflicts of Interest

The authors declare no conflicts of interest.

## Patient Consent Statement

No specific consent statement was obtained, as the functional assessment was done as a part of the survivorship program. Additionally, consent was waived in accordance with the Medical Council of India's Code of Medical Ethics (17.17), which permits such waivers for retrospective studies with minimal risks when patient identities are not disclosed. Additionally, it was waived by the ethics committee.

## Synopsis

Retrospective studies have supported the role of surgery for recurrent retroperitoneal sarcomas. This study highlights the oncological outcomes of patients who underwent surgery for multiple (upto 7) recurrences. Our findings support offering surgery for patients with multiple local recurrences.

## Data Availability

The data of the subjects in this study were collected from the electronic medical records of the institution. The authors affirm that all relevant raw data, will be freely available to any scientist wishing to use them for non‐commercial purposes, without breaching participant confidentiality.
